# Membrane microdomains emergence through non-homogeneous diffusion

**DOI:** 10.1186/2046-1682-5-6

**Published:** 2012-04-30

**Authors:** Hédi A Soula, Antoine Coulon, Guillaume Beslon

**Affiliations:** 1 Université de Lyon Inserm UMR1060, F-69621, Villeurbanne Cédex, France; 2 Université de Lyon, CNRS INSA-Lyon, LIRIS, UMR5205, F-69621, France; 3 EPI BEAGLE, INRIA, F-69603, France; 4 Laboratory of Biological Modeling, NIDDK, NIH, Bethesda, MD, 20892, USA

**Keywords:** Membrane domains, Non-homogeneous diffusion, Individual-based model

## Abstract

**Background:**

In the classical view, cell membrane proteins undergo isotropic random motion, that is a 2D Brownian diffusion that should result in an homogeneous distribution of concentration. It is, however, far from the reality: Membrane proteins can assemble into so-called microdomains (sometimes called lipid rafts) which also display a specific lipid composition. We propose a simple mechanism that is able to explain the colocalization of protein and lipid rafts.

**Results:**

Using very simple mathematical models and particle simulations, we show that a variation of membrane viscosity directly leads to variation of the local concentration of diffusive particles. Since specific lipid phases in the membrane can account for diffusion variation, we show that, in such a situation, the freely diffusing proteins (or any other component) still undergo a Brownian motion but concentrate in areas of lower diffusion. The amount of this so-called overconcentration at equilibrium issimply related to the ratio of diffusion coefficients between zones of high and low diffusion. Expanding the model to include particle interaction, we show that inhomogeneous diffusion can impact particles clusterization as well. The clusters of particles were more numerous and appear for a lower value of interaction strength in the zones of low diffusion compared to zones of high diffusion.

**Conclusion:**

Provided we assume stable viscosity heterogeneity in the membrane, our model propose a simple mechanism to explain particle concentration heterogeneity. It has also a non-trivial impact on density of particles when interaction is added. This could potentially have an impact on membrane chemical reactions and oligomerization.

## Background

In the classical fluid-mosaic model of membrane, membrane components undergo isotropic random motion akin to Brownian motion [1,2]. In this model, since specific interactions between individual molecules are not considered, resulting equilibrium distribution of components is homogeneous. Thus, the so-called Singer-Nicolson model implicitly assumes that no lateral heterogeneities within the membrane plane exist.

Recently, this picture has considerably evolved towards a non-homogeneous distribution of cell-membranes components [3-5]. Experimental evidence has accumulated that cell membranes, particularly cell surface membranes, are indeed laterally heterogeneous on scales that range from tens of nanometers to a few microns. These heterogeneities are commonly referred to as “microdomains” to contrast them with the membrane macrodomains, the functionally differentiated surfaces of epithelial and other morphologically polarized cells. More evidence points towards the fact that some of these domains are enriched in various lipids such as cholesterol. These are sometimes called lipid rafts [6].

The organization of membranes into microdomains can biologically be interesting because microdomains could strongly affect membrane functions as interacting species are likely to be in higher concentrations in the domains. Indeed a wide collection ofmembrane proteins involved in such processes have been shown to colocalize with rafts. They are thought to play an important role in various cellular processes such as trafficking and signaling to cite but a few [7,8]. In addition, perturbations (like disruption by cholesterol removal) have important effects on cell responses compared to control [9,10]. Surprisingly some recent works even suggest that the non-raftparts of the membrane are protein free [11]. It has been reported that membrane proteins with at least one transmembrane domain or with a hydrophobic modification are enriched in lipid rafts [12].

It is not a surprise that various models have emerged to explain the existence and stability of membrane heterogeneity [13]. In particular, membrane protein diffusion alteration has been extensively studied in the context e.g oftrapping systems [14-16], specific interactions [17-19] or crowding [20,21]. Membrane-skeleton fence or membrane-skeleton corralling models have also been proposed. Transmembrane proteins protrude into the cytoplasm and, in this model, their cytoplasmic domains collide with the membrane skeleton, inducing temporary confinement of the transmembrane proteins within the membrane-skeleton mesh [22-24]. The transmembrane proteins then hop to an adjacent compartment. This model was supported by Monte Carlo simulation results [25].

The latter model therefore rests on the assumption that rafts are well defined stable structure with mixed effects on diffusion. Indeed constrained diffusion within bounds imply an increase in concentration only temporarily since outbound protein have low probabilities to come back. In addition several other works suggest a more complicated picture [26]. Rafts (domains) are not well defined entities. Several measurements seem to indicate that some proteins are stably associated over long but finite period of time with discrete domains. These domains can either diffuse across the cell surface [27] or are immobile, such as cell surface caveolae [28]. Contrary to this view is the dynamic assembly. In this model proteins are transiently occupying raft domains and undergo diffusion both inside and outside the raft [29-31].

Being bound to a domain affects the protein diffusivity [32]. The same applies for lipids as their lateral mobility in a liquid-ordered (raft) phase is slower than in a liquid-disordered phase (non-raft) [29,33,34]. Indeed, several studies suggest that proteins and lipids undergo constrained and/or slowed diffusion within rafts [30,31]. However, individual raft proteins do not appear to undergo correlated diffusion with one another [35].

We propose in this article a simple mechanism which leads to the formation of protein/lipid enriched microdomains. This mechanism is based on non-homogeneous diffusion (NHD). Indeed, many structural constituents of the membrane can alter its viscous (or diffusion) properties. As such, a membrane cannot be approximated anymore by a simple 2D manifold with a constant diffusion but rather should be described by a diffusion profile: a space (position) dependent diffusion function. The membrane composition is therefore by itself a source of heterogeneity in the displacement of trafficking proteins.

We will assume throughout this paper that the cell membrane has a non-spatially constant diffusion tensor that is temporally stable within the timeframe of the diffusion. These assumptions allow a greater generality since one never needs to assume any structurally stable component to define a domain. However we will not address the underlying mechanism behind such diffusion variation and simply assume it exists. Recently, several works have provided plausible mechanisms for viscosity alterations [36-39].

By solving the corresponding equation of motion, we show that the diffusion profile relates simply to the equilibrium concentration profile. In the case of punctual particles without interaction we show that this relation is extremely simple and we give a closed form of the equilibrium. In some cases we are able to provide a closed form for the whole solution and derive a FRAP (Fluorescence Recovery After Photobleaching) estimation of the diffusion in such conditions.

We expand the model by performing simulations with non-punctual interacting particles. We show that the classical phase transition observed for this kind of system is altered by non-homogeneous diffusion – namely resulting in a shift in the transition diagram. This shift allows for extremely high concentration in the slow zones – much higher than for non-homogeneous diffusion or interaction separately – as well as higher clustering properties.

## Methods

### Continuous model

In this first model, membrane particles are independent, punctual and subjected to Brownian motion. The membrane is either a one or two dimensional manifold with periodic boundary conditions. This imposes periodic boundary conditions on the equations of motion.

For simplicity’s sake, we first derive the model and equations for the 1D case (equations for the 2D case are provided afterward using another method). Therefore, we consider the membrane as a segment of length *2 L* centered on zero ([–*L*; *L*]); when a particle crosses one of the boundary it will appear at the other side). We assume that these particles undergo a classical Brownian motion in the position space (i.e. overdamped regime). To describe membrane heterogeneity, the diffusion coefficient depends on the position of the particle on the membrane. This so-called multiplicative noise can be efficiently modeled, in the 1D case, with the Stratonovich formalism yielding the following stochastic differential equation

(1) $$dX_{t}=D(X_{t})\circ dZ$$

Here *X*_*t*_ describes the position of the molecule at time *t* in the segment [–*L*; *L*], *dZ/dt* is the classical Brownian noise (with zero mean and unit variance) and D a nonnegative *2L*-periodic function. Here D is thespace-dependent standard deviation of the noise. Then, the actual diffusion (denoted as D) is equal to $D\left(x\right)=D^{2}\left(x\right)$. We will suppose that D is continuously differentiable and > 0 everywhere.

By setting *ρ*(*x,t*) the probability density function of a particle, we obtain the associated Fokker-Planck equation [40] derived from Eq. 1:

(2) $$\frac{\partial \rho (x,t)}{\partial t}=\frac{1}{2}\frac{\partial }{\partial x}\left(D(x)\frac{\partial }{\partial x}\left[D(x)\rho (x,t)\right]\right)$$

Looking for continuous and differentiable solutions, boundary conditions emerge naturally from the *2 L* periodicity. Namely, *ρ*(*L,t*) = *ρ*(–*L,t*) and ${\frac{\partial \rho (x,t)}{\partial x}|}_{L}={\frac{\partial \rho (x,t)}{\partial x}|}_{-L}$ for all *t ≥* 0. Moreover, fluxes of particles are opposed on each side of the border. Writing this condition leads to ${\frac{\partial }{\partial x}\left[D(x)\rho (x,t)\right]|}_{-L}=-{\frac{\partial }{\partial x}\left[D(x)\rho (x,t)\right]|}_{L}$.

The solution of Eq. 2 can be found at equilibrium, yielding the constant flux *J*:

(3) $$J=D(x)\frac{\partial }{\partial x}\left[D(x)\rho (x)\right]$$

The boundary conditions impose *J*(–*L*) = –*J*(*L*) = *J* = 0 and consequently

(4) $$\rho (x)=\frac{\Omega }{D(x)}$$

with $\Omega ={\left[\int_{-L}^{L}\;\frac{dx}{D(x)}\right]}^{-1}$ So, up to a normalization constant, equilibrium density is the inverse of the square root of the diffusion coefficient (or equal to the square root of viscosity).

This result remains the same for 2D (or any higher dimension): Assuming a non-homogeneous brownian motion in higher dimension via

(5) $$\left(\begin{array}{c} dX_{t}\\ dY_{t} \end{array}\right)=\left(\begin{array}{cc} D(x,y) & 0\\ 0 & D(x,y) \end{array}\right)\circ \left(\begin{array}{c} dZ_{1}\\ dZ_{2} \end{array}\right)$$

in the square [–*L*; *L*]^2^ and *dZ*_*i*_*/dt* (*i* = 1,2) being the classical Brownian noise (with zero mean and unit variance). The Fokker-Planck equation for the density function *ρ*(*x,y*) will be

(6) $$\frac{\partial \rho (x,y,t)}{\partial t}=div(D\nabla (D\rho ))$$

with the boundary conditions

(7) $$\begin{array}{c} \frac{\partial }{\partial x}{\left[D(x,y)\rho (x,y,t)\right]}_{{|}_{\pm L,y}}\\ =\frac{\partial }{\partial x}{\left[D(x,y)\rho (x,y,t)\right]}_{{|}_{x,\pm L}}=0 \end{array}$$

for all *t ≥* 0. To solve Eq. 6, we multiply by Dρ and integrate with respect to both *x* and *y* to obtain $\frac{1}{2}\frac{\partial }{\partial t}\int \int D\rho^{2}dxdy=\int \int D\rho div(D\nabla (D\rho ))dxdy$ since $\frac{\partial }{\partial t}\rho^{2}=\frac{1}{2}\rho \frac{\partial \rho }{\partial t}$ An integration by parts yields $\begin{array}{l} \int \int D\rho div(D\nabla (D\rho ))dxdy=\int_{{\left[-L;L\right]}^{2}}\;D^{2}\rho \nabla (D\rho )\\ -\int \int \nabla (D\rho )D\nabla (D\rho )dxdy \end{array}$. The first integral on the right hand side of the previous equation is taken over the square [–*L*; *L*]^2^ and vanishes due to toric boundary conditions (Eq. 7), yielding

(8) $$\frac{1}{2}\frac{\partial }{\partial t}\int \int D(x,y)|\rho {|}^{2}dxdy=-\int \int D|\nabla (D\rho ){|}^{2}dxdy$$

Since D is > 0 everywhere the left hand side of Eq. 8 is a decreasing function and its derivative must reach zero at equilibrium.

This equilibrium therefore verifies

(none) $$\int \int D|\nabla (D\rho ){|}^{2}dxdy=0$$

meaning the gradient of Dρ is zero everywhere. That is

(9) $$\rho (x,y)=\frac{\Omega }{D(x,y)}$$

The same result holds for any dimension: any diffusion over a non-constant diffusion profile yields, at equilibrium, to a non constant concentration namely its inverse. Note that as it should be, in the case of homogeneous diffusion ($D(x)\equiv \sqrt{D_{0}}$) the equilibrium density (*ρ*(*x*) ≡ (2 *L*)^–1^) is independent of the diffusion coefficient *D*_0_.

As a consequence zones of slow diffusion (or high viscosity) will tend to gather more particles at equilibrium than any faster zones. Note that we used the Stratonovich formalism to describe non-homogeneous brownian motion of particles. This formalism make assumptions on the diffusion at the microscopic scale [41]. With other assumptions, we could have derived the same equations using the Ito formalism to obtain a slightly different result.

Briefly: the associated Fokker-Planck would have been:

(10) $$\frac{\partial \rho (x,y,t)}{\partial t}=div(\nabla (D^{2}\rho ))$$

yielding an equilibrium distribution

(11) $$\rho (x,y)=\frac{\Omega }{D^{2}(x,y)}$$

The main results hold in a stronger way (due to the square) in any dimensions. In the continuous model, all simulations described below have also been done using the Ito formalism for the random motion scheme. All the results at equilibrium described below hold qualitatively in the Ito formalism but in a stronger way quantitatively. Thus, to stress our point, we will focus on the ‘weaker’ form of our results and remain in Stratonovich formalism throughout the rem`aining of the paper.

The previous results hold for equilibrium distribution only. General transient solutions can be obtained for the one dimensional case by solving Eq. 2 entirely. Let $\tilde{x}(x)=\int_{-L}^{x}\;\frac{du}{D(u)}$ and $p(\tilde{x},t)=D(x)\rho (x,t)$ Eq. 2 becomes

(12) $$\frac{\partial p}{\partial t}=\frac{1}{2}\frac{\partial^{2}p}{\partial {\tilde{x}}^{2}}$$

which is exactly the classical diffusion equation. One can immediately see that the same boundary conditions apply to *p* by replacing *x* by $\tilde{x}$. Equilibrium solution for classical diffusion on a circle is a constant density. So $D(x)\rho (x,t)=p(\tilde{x},t)=\Omega$. In addition, we obtain the transient solutions (on a circle) by replacing *x* by $\int_{-L}^{x}\;\frac{du}{D(u)}$ in the general solutions of the classical diffusion. Note that depending on the initialconditions, particles can take a longer time to reach equilibrium in the slow diffusing zones. Therefore, transiently it may happen that zones of slow diffusion will gather less particles than in faster zones.

On biological membranes, transient solutions are impossible to measure. The classical solution is to estimate them indirectly via imaging techniques such as Fluorescence Recovery After Photobleaching (FRAP). Bleaching particles amounts to create a new initial distribution *ρ*(*x*,0) = *g*(*x*). Particles inside the bleaching are removed (actually bleached) while the others are untouched: *g*(*x*) = 0 for |x|<r (for a bleaching beam of radius *r* centered on zero). In that case, the classical approximation of the half-time to recovery [42,43] is

(13) $$\tau_{r}=\frac{\gamma r^{2}}{D}$$

where *γ* is a constant. Replacing *r* by $\int_{-r}^{r}\;\frac{du}{D(u)}$ and using $D(r)=D^{2}(r)$, we can account for an heterogeneous diffusion dependent half-time recovery.

(14) $$\tau_{r}=\gamma {\left(\int_{-r}^{r}\;\frac{du}{D(u)}\right)}^{2}=\gamma {\left(\int_{-r}^{r}\;\frac{du}{\sqrt{D(u)}}\right)}^{2}$$

Equation 14 indicates that the time constant of the recovery is proportional to the square of the spatial average of the *inverse* of the square root of diffusivity. Using this equation, one can, theoretically, extracts the diffusion profile from FRAP data [44]. Unfortunately, for a general diffusion profile, no such transient solution can be found for higher dimensions.

At this point, we can already make some useful predictions on membrane particle distribution. Indeed, some membrane components are known to alter membrane viscosity. Our model suggests that, around components that increase viscosity, diffusing particles will be in higher concentration – overconcentration – compared to faster zones. A good candidate could be cholesterol: Indeed, cholesterol is found in high amount on virtually all mammal cells and is known to rigidify membranes and cholesterol-enriched membranes display lower diffusion coefficient (by around one order of magnitude) [45,46]. In this case our model predicts that membrane cholesterol islands (patches) are *originating* membrane domains. In other words and without any more assumptions, stable cholesterol domains should imply by means of slow diffusion a protein-riched domain.

In addition, our model estimates this domains to concentrate more particles in proportion to the slowing. Indeed this overconcentration is simply proportional to the square root of the ratio of diffusion. For example, diffusion an order of magnitude slower should provide slow domains with around three times more particles.

Conversely, when cholesterol concentration is low enough or high enough for the membrane to exhibit an approximatively constant diffusion profile, our model predicts no domain.

### Force field interactions

This first model is neglecting some crucial features of membrane diffusing particles. As such, our model assumes independent particles that never collide nor hinder each other and that cannot interact. But both crowding and particle interactions are known to yield inhomogeneous particle distribution. We expand the previous model to take into account steric hindrance and possible particle interactions (e.g. protein-protein interactions) [47].

To study the combined effect of non-homogeneous diffusion and particle interaction, we used 2D particle simulations. As such, particle interactions must be modeled at the same scale as the diffusion itself: the mesoscopic scale. In order to do so, particles were subjected to non-homogeneous Brownian motion and short-distance interactions [48,49]. Assuming there are *N* particles, we introduce a new equation of motion for a particle *j* as:

(15) $$\begin{array}{ll} \left(\begin{array}{c} dx_{j}\\ dy_{j} \end{array}\right) & =D^{2}(x_{j},y_{j})\sum_{i=1,i\neq j}^{N}\;f(r_{\mathrm{ij}})u_{\mathrm{ij}}\\ & +D(x_{j},y_{j})\left(\begin{array}{c} dZ_{x}\\ dZ_{y} \end{array}\right) \end{array}$$

where *u*_*ij*_ is the unitary vector of the semi-line starting from particle *i* to particle *j*, *r*_*ij*_ being the distance between the two particles. The interaction force *f* is derived from the 6 – 12 Lennard-Jones empirical potential accounting for van der Waals interactions:

(16) $$P(r)=\in \left({\left(\frac{\sigma }{r}\right)}^{12}-2{\left(\frac{\sigma }{r}\right)}^{6}\right)$$

the force *f* itself being the opposite of the gradient of the potential $f(r)=-\frac{dP(r)}{dr}$. The two parameters *∈* and *σ* describe the shape of the interaction. For a given *σ* increasing *∈* increases the strength of the interaction (as well as its range) and clusters of particles appear. The average size ofclusters with increasing *∈* yields a phase transition from *N* clusters of size 1 to one cluster of size *N*[50,51].

However, in the case of non-homogeneous diffusion, the phase transition will be shifted to lower interaction strength in the slower zones. As such, fast zones will act as a particle provider (well) and slow zones as particles sink. We expect therefore to obtain in the slow zones a higher over-concentration compared to the fast zones but also a higher degree of clustering. This over-concentration should be higher than both effects (interaction and non-homogeneous diffusion) taken separately.

## Results and discussion

### Continuous model

We present in this section the results obtained via simulation of particles that undergo equation of motion such as Eq. 1. We used the Milstein numerical schema for the Stratonovich formalism [52],

(17) $$\begin{array}{ll} X_{i}(t_{k+1})= & \sqrt{\Delta t}D(X_{i}(t_{k}))Z\\ & +\frac{\Delta t}{2}D(X_{i}(t_{k}))D^{\prime }(X_{i}(t_{k}))Z^{2} \end{array}$$

Where *Xi* (*t*) is the position of particles *i* at time *t* (*t*_*k*_ = (*k +* 1)Δ*t*) and D and $D^{\prime }$ are the diffusion profile and its gradient respectively. Various diffusion profiles were tested as well as different number of particles. For the 1 dimensional experiment 100,000 particles were used whereas 1,000,000 were used for the 2 dimensional case. We tested various time step and took the highest value (Δ*t* = 0.01)) that yielded no change in equilibrium.

**Figure 1:**
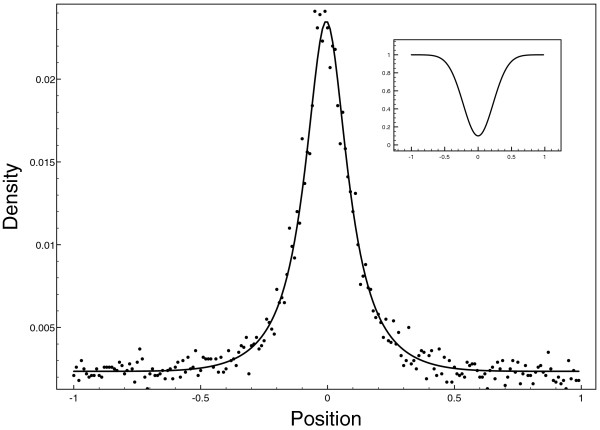
**Results for 1 d experiment.** Simulation (dots) versus theoretical (plain line) distribution of particles that undergo non-homogeneous diffusion. The diffusion profile is a simple inverse gaussian function (in inset) with equation: $D(x)=1-0.9\text{exp}(-\frac{x^{2}}{\sigma^{2}})$ and *σ* = 0.1 (theoretical:plain line, simulations: triangles).

Equilibrium distribution behaved as expected. The first set of experiments is for the one dimensional case where we test various diffusion profiles (continuously differentiable *2 L*-periodic). As an example, we present two of them: In one case, Figure 1, the diffusion profile is a Gaussian function $D(x)=D_{0}(1-d\text{exp}(-x^{2}/\omega )$ with *D*_0_ = 1, *d =* 0.9 and *ω* = 0.1 (displayed in inset). The theoretical distribution Ω/D(x) and the simulated particle distribution are depicted by a plain line and dots respectively.

**Figure 2:**
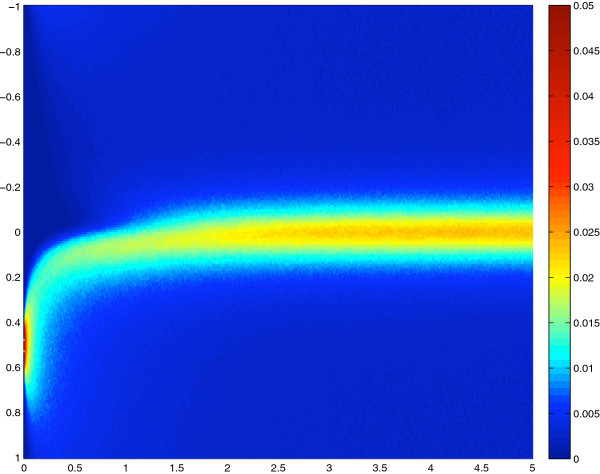
**Transient distribution.** Simulation of *ρ*(*t,x*) for t ∈ [0:5] and x ∈ [–1:1]. Setting *ρ*(0,*x*) = *δ*(*x*–0.5) as the initial condition. The diffusion profile is as in Figure 1 and therefore the distribution obtained near *t =* 5 is the same as the one at equilibrium described in Figure 1.

Transients for this simulation are displayed on Figure 2 as *ρ*(*t,x*) for 0 ≤ *t ≤* 10 and *x*∈[–1:1]. The initial condition is *ρ*(0,*x*) = *δ* (*x* – 0.5) a Dirac function centered on 0.5.

In this case, due to the slow diffusivity the concentration at the peak of viscosity *ρ*(*t*,0.0) is the lowest value for a long period: transiently the slow zone is under populated.

**Figure 3:**
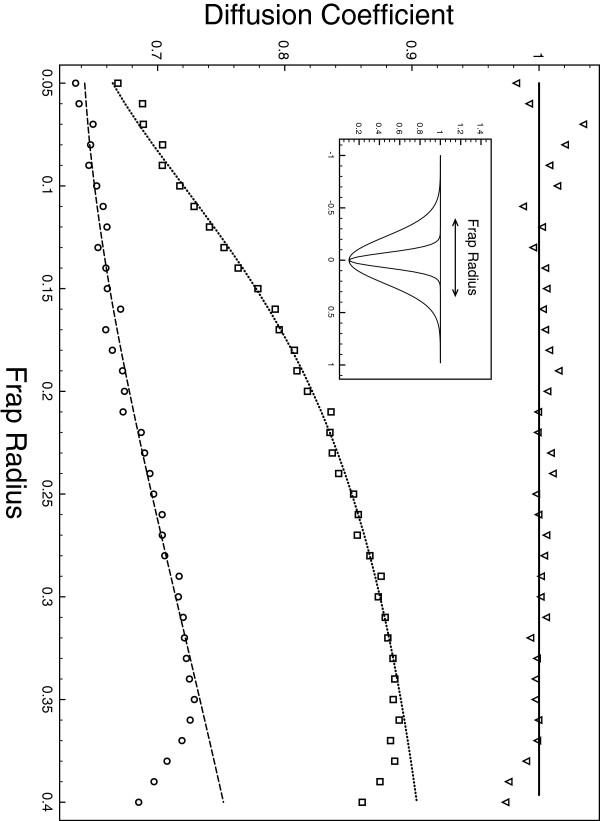
**FRAP experiment.** Results for FRAP experiments compared to theoretical prediction. Three experiments with different diffusion profiles are depicted. *D* = 1 (theoretical:plain line, simulations: triangles) and two gaussian profile $D(x)=1-0.9\text{exp}(-\frac{x^{2}}{\sigma^{2}})$ with σ = 0.01 (middle, theoretical: dotted line, simulations: squares) and σ = 0.1 (bottom, theoretical: dashes line, simulation: circles).

In addition, we simulated FRAP experiments and compared the diffusion coefficients computed through simulation to the theoretical ones as a function of the FRAP radius *r* (the FRAP experiment was centered on the slowest diffusion point). Figure 3 shows the results for three experiments, one classical homogeneous diffusion with D(x)=1 and two NHD with a centered gaussian $D(x)=1-de^{-x^{2}/\omega }$ with *d =* 0.9 with *ω =* 0.1 and *ω =* 0.01 (shown in inset). Triangles, squares and circles are diffusion coefficient computed from the particle simulation for the cases $D\left(x\right)=1$, D=D(x) with *ω* = 0.1 and *ω* = 0.01 respectively. The lines are their theoretical counterparts computed using Eq. (14).

**Figure 4:**
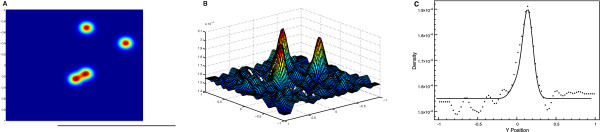
**Results for 2 d experiment. A**) The diffusion profile in light spectrum (toward red is slower) for 4 randomly “cholesterol” islands. **B**) 2 d histogram of simulated particles positions at equilibrium. **C**) Comparison with theoretical prediction for a cross section (*x* = 0.25). This section is represented by a black line on the heightmap and a white line on the 2 d histogram.

Next, we present the results of NHD on a two-dimensional torus. The diffusion profile consists of 4 randomly positioned Gaussian patches centered on (*x*_*i*_,*y*_*i*_) ∈ [–*L; L*]^2^ with 1 ≤ *i* ≤ 4 and $D_{i}(x,y)=D_{0}(1-dexp(-(({\left(x-x_{i}\right)}^{2}+{\left(y-y_{i}\right)}^{2})/\omega )$ with *D*_0_ = 1, *d* = 0.9 and *ω* = 0.1. The overall diffusion profile is the average $D(x,y)=\frac{1}{4}\sum_{i=1}^{4}\;D_{i}(x,y)$. Figure 4 shows the diffusion profile (light spectrum heightmap, Figure 4A) and the 2D histogram of the resulting equilibrium distribution of particles (Figure 4B). The last panel (4C) compares a cross-section (*x* = 0.2 is constant) of theoretical and simulated distributions. This cross-section can be visualized as a black line on the heightmap of Figure 4A and as a white line on Figure 4B.

In both one and two dimensional cases, the equilibrium distribution of particles that undergo non-homogeneous diffusion matches the theoretical prediction. Slower zones concentrate more particles than the others. In addition this over-concentration isproportional to the amount of slowing.

### Force field interactions

Simulations were performed for the particles with interaction. To take into account steric hindrance, the numerical schema (Eq. 17) was modified by adding an interaction strength vector Δ*tf*(*X*_*i*_(*t*)).

Collision detection was included to allow a simple crowding experiment (i.e *∈* = 0 in Eq. 16). We present the following experiment: On a 2D square (with toric boundary conditions) we insert a squared slow patch (on the upper leftcorner) where diffusion D is $r=D_{0}/D$ times smaller than the diffusion ($D_{0}$) in the remaining of the space: a “cholesterol” patch. Another square of the same surface away from the cholesterol patch is used as control patch. Numbers of particles are counted in both patches at equilibrium and normalized by the total number of particles and the size of the patch.

**Figure 5:**
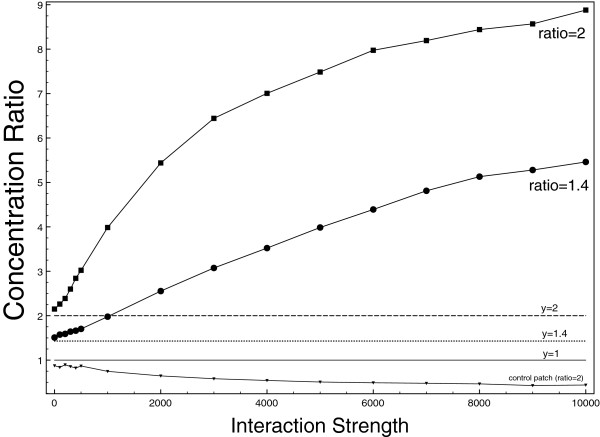
**Particles interactions results.** Concentration of particles in the “cholesterol” patch for two diffusion ratios $r=D_{0}/D$ with r = 2 (black square) and r = 1.4 (black circle). Plain line is the control concentration with D=1. Dashed line is the theoretical NHD concentration.

**Figure 6:**
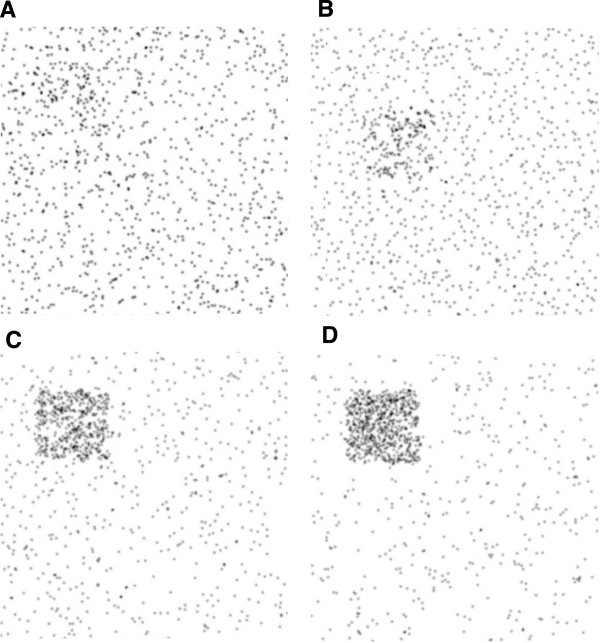
**Particles maps.** Position map of particles for **A**) ***ε*** = 0 and r = 2, **B**) ***ε*** = 500 and r = 2, **C**) ***ε*** = 4000 and **D**) ***ε*** = 10000. Note that the actual values of *ε* are scaled down by 10^–12^. See also supporting Additional file 1: Video S1, Additional file 2: Video S2, Additional file 3: Video S3 and Additional file 4: Video S4 for *r*∈{2,10} and *∈*∈{0,1*e5*}.

Assuming cholesterol diffusion is reduced by around one order of magnitude [53-56], those numbers are displayed on Figure 5 for two conservative (i.e higher) values of the diffusion ratio $r=D_{0}/D=2$ (black square) and *r =* 1.4 (black circles). The dashed lines are the theoretical predictions for the concentration in the patch in case of punctual particles (that is *y = r*). For low interaction strength, the punctual particles prediction remains a good approximation. However when the interaction increases, concentration of particles in the cholesterol patch increases dramatically while there is no such increase in the control patch. On Figure 6 screenshots of particles positions are displayed (*r =* 0.5) for *∈* = 0 (A), *∈* = 500(B), *∈* = 4000(C) and *∈* = 10000(D) – see also supporting videos.

As expected, the overconcentration effect due to non-homogeneous diffusion is dramatically increased via particles interaction. As Figure 5 shows it, a great portion of the particles is concentrated in the cholesterol patch. In this extreme case, the patch is virtually composed of one big cluster of particles in interaction whereas there are no clusters in the remaining of the space.

**Figure 7:**
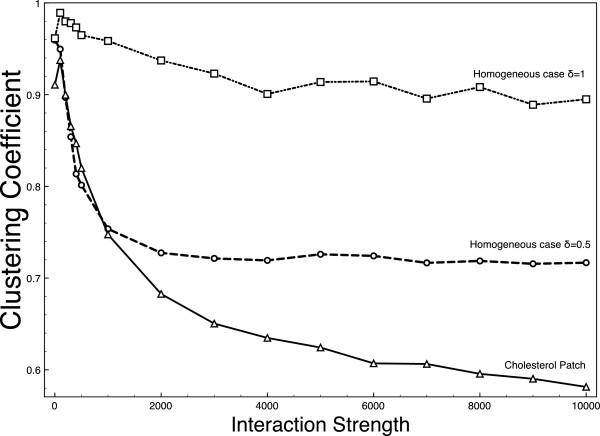
**Effects on clustering.** Clustering coefficient (see text for definition) as a function of the interaction strength ***ε*** for control D=1 (squares), slow experiment D=0.5 (circles) and cholesterol square patch withration r = 2 (triangles).

To numerically assess this situation, we compute the clustering coefficient as a function of the interaction strength: *c*(*f*) = *N*_*c*_*/N*_*i*_ where *N + N*_*c*_ is the number of clusters and *N*_*i*_ is the number of particles involved. Clusters were computed by creating a graph of particles whose nodes are the particles themselves and link between two nodes exists if the distance between their centers is below *ar*_0_ (*a* = 2.7 was chosen a bit higher than *σ* = 2.5). A value close to 1 indicates no clustering while a more pronounced clustering yields smaller values. Results are displayed in Figure 7. The values are computed in three situations: in the first two, diffusion is homogeneous with diffusion D=1 (squares) and D=0.5 (circles). The remaining case (triangles) is the one with a cholesterol patch with ratio *r = 2* ($D_{0}=1$ as in the previous experiments). In all cases, the clustering coefficient decreases with the interaction strength and thereforeclusters appear (one needs higher interaction strength for the case D=1). Moreover, the lower the viscosity the deeper the clustering. However, for high enough interaction strength, the cholesterol patch does not behave as an homogeneous slow zone: whereas cluster sizes seem to reach a plateau in the slow diffusion experiment, clusters were bigger in the cholesterol patch.

**Figure 8:**
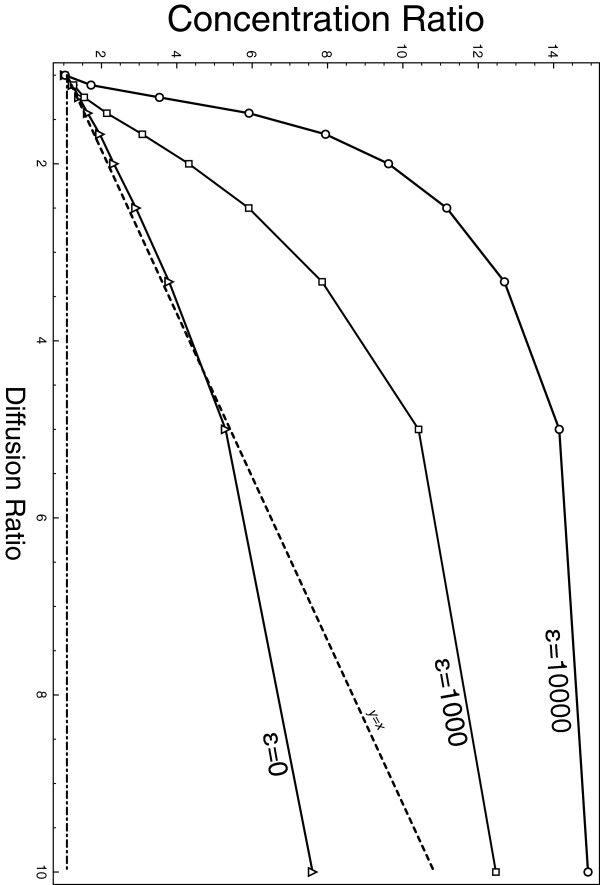
**Diffusion value dependence.** Concentration ratio as a function of the ratio of diffusion for three interactions strength ***ε*** = 0 (triangles), ***ε*** = 1000 (squares), and, ***ε*** = 10000 (circles). Bold dashed line is the theoretical prediction of concentration for the case of no interaction (punctual particles) *y = x*. To guide the eye the line *y* = 1 is displayed.

To mimic cholesterol effect, we deliberately chose diffusion ratios $r^{2}=D_{0}^{2}/D^{2}=D_{0}/D$ to be conservative i.e lower than one order magnitude of the diffusion ratio (*r*^*2*^ ranges from 2 to 4 instead of 10) in almost all experiments [53-56]. However, for completeness’ sake, we finally test several values of diffusion ratios. Results are summed up in Figure 8 for three values of the interaction strength (∈) and for ratios going from *r = 1* (no slowing) to *r* = 10. Typical extremal dynamics are available on the supporting Additional file 1: Video S1, Additional file 2: Video S2, Additional file 3: Video S3 and Additional file 4: Video S4.

These results first confirm the predictions for punctual particles. With the parameters tested and as shown on Figure 8, the crowding only experiment did not differ much from the predictions. In addition, we show that particle interactionsin the form of Lennard-Jones potential can strongly modify both the magnitude and the nature of the resulting overconcentration. Around conservative values for diffusion, the so-called cholesterol patch can gather up to 9 times more particles than expected for an homogeneous medium. In addition, these particles tend to be more in clusters than any other situation. Our results can only be obtained and explained by combining both NHD and particle interactions. The cholesterol patch – the domain – spontaneously gathers particles and increases the stability of their interactions.

## Conclusion

Cell membrane can display a wide variety of heterogeneity in its physical properties, viscosity and constituents composition. Our model describes the possible emergence of what we call a minimal membrane domain based on only one feature of the membrane, namely its local viscosity. We show that, everything else being equal, domain with high viscosity tends to gather statistically a larger proportion of the diffusive particles. Particles are not trapped within these domains but merely tend to spend more time in them. In addition, we show that the ratio of concentration of particles in and out domain is proportional to the square root of the inverse ratio of their local diffusion. As such, protein free membrane zones are seen as a product of high diffusivity and not from forbidding constraints – contrary to the hypothesis developed in [11] – and in accordance with the results found in [26]. Provided we assume that viscosity is indeed non constant for relevant time scales, this provides an extremely simple and parsimonious explanation for protein-enriched membrane domains.

Moreover, while this model is fairly general, we emphasize the particular role of cholesterol. Cholesterol is ubiquitous in cell membranes and its influence on membrane diffusion has been well documented. Therefore, it is not surprising, from our point of view, that cholesterol has been found in higher quantity in virtually all microdomains exhibited so far. For many authors, its presence even characteries membrane domains [3,11]. Our model shows that gradient in cholesterol concentration leads naturally to protein domains. This property depends only on the relative diffusion between cholesterol-rich zones and the remaining of the membrane.

Looking for experimental results, authors have reported the impact of temperature and cholesterol depletion on membrane heterogeneities. Cholesterol removal should decrease the colocalization of membrane components as in [57,58]. In addition, cooling decrease the diffusion ratio between membrane with and without cholesterol. For low temperature it even flips: our model predicts no colocalization or domains in the case of low temperature as it has been reportedin [58,59].

Important diffusion variation leads not only to a quantitative particles concentration variation but also to a qualitative variation. Protein interactions alter significantly the particles concentration landscape when combined with NHD. We show that cluster of particles are more stable in slower zones and appear for lower values of interactions. Our model predicts that domains of slow diffusion will alter affinity interactions. That is, for example oligomerization will happen preferentially inside these domains. This latter prediction has been observed on model membrane where modifications of the cholesterol content trigger oligomerization [60]. Thus, chemical reactions will have their equilibrium shifted in these zones, revealing a potential function for membrane microdomains.

## Competing interest

The authors declare that they have no competing interests.

## Authors’ contributions

HAS designed the study, performed the simulations, analyzed the simulation data and drafted the manuscript. AC and GB conceived the study, analyzed the data and drafted the manuscript. All authors read and approved the final manuscript.

## Supplementary Material

Additional file 1 **Particle evolution for *r* = 2 and no interactions (Є = 0).**Evolution under non-homogeneous diffusion of 1,000 particles (depicted in green) for 1000 time step. A square slow patch on the upper left corner has a diffusion ratio of 2 and particles interaction is limited to collision. This is the typical NHD simulations when, at equilibrium, there will be twice more particles in the slow patch.
Click here for file

Additional file 2 **Particle evolution for *r* = 2 and no interactions (Є = 10000).**Evolution under non-homogeneous diffusion of 1,000 particles (depicted in green) for 1000 time step. A square slow patch on the upper left corner has a diffusion ratio of 2 and particles interaction is set to an intermediate level Є = 10000. For this important level of interactions, clusters appear everywhere but they are always more pronounced in the slow patch.
Click here for file

Additional file 3 **Particle evolution for *r* = 10 and no interactions (Є = 0).**Evolution under non-homogeneous diffusion of 1,000 particles (depicted in green) for 500 time step. A square slow patch on the upper left corner has a diffusion ratio of 10 and particles interaction is limited to collision. The important amount of slowing allows for an important overconcentration (that will ultimately be 10 fold).
Click here for file

Additional file 4 **Particle evolution for *r* = 10 and no interactions (Є = 10000).**Evolution under non-homogeneous diffusion of 1,000 particles (depicted in green) for 500 time step. A square slow patch on the upper left corner has a diffusion ratio of 10 and particles interaction strength is set to 10000. In this last experiment,the over-concentration appears first on the border of the patch. The difference in the phase transition is extremely more pronounced in the slow patch.
Click here for file
